# Elevated risks of death for diabetes mellitus and cardiovascular diseases in Italian AIDS cases

**DOI:** 10.1186/1742-6405-7-11

**Published:** 2010-05-24

**Authors:** Diego Serraino, Silvia Bruzzone, Antonella Zucchetto, Barbara Suligoi, Angela De Paoli, Simona Pennazza, Laura Camoni, Luigino Dal Maso, Paoli De Paoli, Giovanni Rezza

**Affiliations:** 1 Unit of Epidemiology and Biostatistics, Centro di Riferimento Onocologico, IRCCS, Aviano, Italy; 2 Direzione centrale per le statistiche e le indagini sulle istituzioni sociali, Servizio Sanità e Assistenza ISTAT, Rome, Italy; 3 Dipartimento Malattie Infettive, Istituto Superiore di Sanità, Rome, Italy; 4 Direzione Scientifica, Centro di Riferimento Onoclogico, IRCCS, Aviano, Italy

## Abstract

After the introduction of highly active antiretroviral therapies (HAART), an increased incidence of insulin resistance, diabetes mellitus (DM), and cardiovascular diseases has been described. The impact of such conditions on mortality in the post-HAART era has been also assessed in various modes in the literature. In this paper, we report on the death risks for DM, myocardial infarction, and chronic ischemic heart diseases that were investigated among 9662 Italian AIDS cases diagnosed between 1999 and 2005. Death certificates reporting DM, myocardial infarction, and chronic ischemic heart diseases were reviewed to identify the underlying cause of death, and to compare the observed numbers of deaths with the expected ones from the sex- and age-matched, general population of Italy. Person-years at risk of death were computed from date of AIDS diagnosis up to date of death or to December 31, 2006. Standardized mortality ratios (SMR) and their 95% confidence intervals (CI) were computed. DM and cardiovascular diseases were the cause of death for 43 out of 3101 deceased AIDS cases (i.e., 1.4% of all deaths). In comparison with the general population, the risks of death were 6.4-fold higher for DM (95% CI:3.5-10.8), 2.3-fold higher for myocardial infarction (95% CI:1.4-3.7) and 3.0 for chronic ischemic heart diseases (95% CI: 1.5-5.2).

## Findings

HIV-infected people are at increasing risk of developing several non-AIDS defining illnesses, including diabetes mellitus (DM) and cardiovascular diseases [1-3]. Traditional risk factors (such as cigarette smoking, ageing, obesity, and viral co-infections) and duration of HIV infection are considered responsible of their elevated frequency, though they have also been associated with adverse effects of antiretroviral treatments [1,4-6]. Several studies have evaluated the incidence of DM and cardiovascular diseases in HIV-infected persons in the era of highly active antiretroviral therapies (HAART), and their impact as causes of death [7-10].

By taking advantage of the population-based data used for assessing post-AIDS survival [11], we estimated the risk of death for DM, myocardial infarction, and chronic ischemic heart diseases among people with AIDS diagnosed between 1999 and 2005. The original study design and the main characteristics of study subjects were previously described [11]. Briefly, in Italy AIDS cases are diagnosed according to the 1993 revised European AIDS definition [12], and they are compulsorily reported to the national AIDS registry (RNAIDS), a comprehensive surveillance system formerly described in detail [13]. Under-reporting of people with AIDS (PWA) has been estimated at about 5% [14], whereas the vital status of PWA is not routinely kept up-to-date. The updated vital status of PWA was sought for in the Italian Mortality Database at the Italian National Institute of Statistics through a record linkage procedure. Data regarding PWA diagnosed in Italy from 1999 and 2005 were linked with data concerning the 4,420,498 deaths occurred between 1999 and 2006. After excluding non Italian citizens, pediatric cases, PWA diagnosed solely at autopsy who were residents in provinces where information on names were not available on deaths certificates, 9662 Italian adult PWA constituted the study population. Of these PWA, 3101 died. Conditions listed in the death certificate were classified as AIDS- or non-AIDS-related based on the presence/absence of an AIDS-defining condition according to the 1993 revised European AIDS definition [12].

Deaths certificates reporting DM, myocardial infarction, and chronic ischemic heart diseases in any position were reviewed by study members to distinguish when one of these conditions was the underlying cause of death (i.e., the disease which initiated the sequence of morbid events leading directly to death) or a contributing one. This process was undertaken to properly compare the observed numbers of DM, myocardial infarction, and chronic ischemic heart diseases as underlying cause of death in PWA with the expected numbers from the sex- and age-matched, general population of Italy. The codification rules of the International Classification of Diseases, tenth revision (ICD-10), were applied.

We took into consideration all ICD-10 codes pertaining to DM (i.e., E10-E14), while for cardiovascular causes of death, we focused on acute myocardial infarction (ICD-10, I21) and chronic ischemic heart diseases (ICD-10, I25), two important and well diagnosed conditions.

Person-years (PY) at risk of death were computed from date of AIDS diagnosis up to date of death or to December 31, 2006. The number of observed deaths due to DM, myocardial infarction, or chronic ischemic heart diseases was divided by the expected one, computed from age and sex specific mortality rates from the Italian general population in the same period. Thus, standardized mortality ratios (SMR) and their 95% confidence intervals (CI) were computed [15].

The 9,662 AIDS cases included in this study summed up to 34,814 PY of follow-up and 3,101 deaths. The most frequent conditions listed in death certificates were hepatic diseases (reported in 31.0% of death certificates), AIDS-associated opportunistic infections (29.2%), other infectious diseases not included in the AIDS definition (24.1%), non-Hodgkin lymphoma (14.6%), and Kaposi sarcoma (4.3%) (data not shown).

DM and cardiovascular diseases were the underlying cause of death of 43 PWA (i.e., they cause 1.4% of all deaths): 14 deaths were caused by DM, 17 by myocardial infarction, and 12 by chronic ischemic heart diseases Table 1. The corresponding SMR were 6.4 for DM (95% CI:3.5-10.8), 2.3 for myocardial infarction (95% CI:1.4-3.7) and 3.0 for chronic ischemic heart diseases (95% CI: 1.5-5.2).

**Table 1:** Standardized mortality ratios of dying from diabetes mellitus, myocardial infarction, or chronic ischemic heart diseases in people with AIDS, according to selected characteristics. Italy, 1999-2006

		Cause of death								
		**Diabetes mellitus**			**Myocardial infarction**			**Chronic ischemic heart diseases**		
		
	**PY**	**Obs**	**Exp**	**SMR** **(95% CI)**	**Obs**	**Exp**	**SMR** **(95% CI)**	**Obs**	**Exp**	**SMR** **(95% CI)**

**Age at AIDS diagnosis** **(n° of cases)**										
<45 (6958)	26389	5	0.36	13.8 (4.4-32)	4	1.97	2.0 (0.5-5.2)	4	0.51	7.8 (2.0-20)
≥45 (2704)	8425	9	1.82	4.9 (2.2-9.4)	13	5.34	2.4 (1.3-4.2)	8	3.50	2.3 (1.0-4.5)
										
**Sex (n° of cases)**										
Male (7511)	26962	14	1.97	7.1 (3.9-11.9)	14	7.01	2.0 (1.1-3.4)	9	3.81	2.4 (1.1-4.5)
Female (2151)	7851	0	0.21	0	3	0.30	9.9 (1.9-29)	3	0.20	14.9 (2.8-44)
										
**HIV-transmission category (n° of cases)**										
Intravenous drug user (4040)	14235	5	0.28	18.2 (5.7-43)	5	1.42	3.5 (1.1-8.3)	4	0.41	9.7 (2.5-25)
Homosexual man (1741)	6512	2	0.61	3.3 (0.3-12.1)	0	2.08	0	3	1.14	2.6 (0.5-7.8)
Heterosexual (3297)	12121	5	1.06	4.7 (1.5-11.1)	10	3.14	3.2 (1.5-5.9)	3	1.99	1.5 (0.3-4.5)
Other (584)	1945	2	0.24	8.5 (0.8-31)	2	0.67	3.0 (0.3-10.9)	2	0.47	4.2 (0.4-15.6)
										
**Total (9662)**	34814	14	2.18	6.4 (3.5-10.8)	17	7.31	2.3 (1.4-3.7)	12	4.01	3.0 (1.5-5.2)

SMR = Standardized mortality ratio; PY = Person Years; Obs = Observed; Exp = Expected; CI = Confidence interval

The risk of death associated to DM was more pronounced in younger (SMR = 13.8) than in older individuals (SMR = 4.9), and it was restricted to men Table 1. An 18-fold higher risk of death for DM was seen among intravenous drug users (IDUs). The risks of death associated with myocardial infarction and with chronic ischemic heart diseases were substantially elevated in women (SMR = 9.9 and SMR = 14.9, respectively), whereas they were of borderline statistical significance in men. A particularly pronounced excess risk was seen for chronic ischemic heart diseases in cases aged less than 45 years at AIDS diagnosis (SMR = 7.8) Table 1. The SMR for chronic ischemic heart diseases was nearly 10-fold higher in IDUs than in the general population Table 1

DM, myocardial infarction, and chronic ischemic heart diseases had a marginal impact (i.e., 1.4%) on mortality of Italian AIDS cases in the post-HAART era. However, the risks of death were remarkably higher than in the corresponding general population. We have used mortality rates in the general population as a bench mark for comparison, because the overall mortality of HIV-infected individuals is increasingly influenced by deaths that would have occurred regardless of HIV infection. Although based on small numbers, differences in the magnitude of SMRs according to sex, age and HIV-transmission category seem to point toward multiple risk factors (e.g., smoking for cardiovascular diseases). We have previously demonstrated, among Italian AIDS cases below the age of 55 years, a nearly 5-fold excess death risk for pancreatic cancer [16]. Such observation is in line with the well established role of DM as a risk factor for pancreatic cancer [17]. With regard to myocardial infarction and ischemic heart diseases, the patterns of risk herein described seem to resemble the one seen, in HIV-infected persons, for smoking-related cancers. Our group has recently shown, in Italian AIDS cases, a 4-fold higher risk in men and a 6-fold higher risk in women of developing lung cancer, an excess risk mainly attributed to a high prevalence of smokers in HIV-infected people [18]. As for lung cancer, HIV-infected people are likely to be at higher risk for other smoking-related diseases, because such a habit is more common among them than in the general population.

Possible limitations of this study should be addressed. With regard to the identification of the underlying cause of death, we adopted the same ICD-10 rules of codification used by the Italian National Institute of Statistics for the general population (i.e., without considering the mention of HIV/AIDS). We acknowledge that the quality of death certificates is low and that the ICD-10 rules may be of difficult application to people with severe immunodeficiency, given that these people are at risk of dying from a multitude of other causes. From a statistical viewpoint, the stratified analyses suffered from the small numbers of observed deaths in several subgroups, whereas the analysis regarding the total number of deaths allowed us a finer computation of SMR. Furthermore, the dataset used for this analysis did not include information on individual risk factors; we were, thus, unable to provide further insights in the reasons for this excess risk. Conversely, completeness was the main advantage of population-based investigations, and for this analysis, we used two databases covering the whole Italian population.

In conclusion, DM and cardiovascular diseases caused a statistically significant excess in the number of deaths of AIDS cases. Understanding the causes of such augmented risks will help reduce mortality from non-AIDS defining illnesses. In this perspective, anti-smoking campaigns may be crucial in making antiretroviral treatments more effective.

## Competing interests

The authors declare that they have no competing interests.

## Authors' contributions

DS, PDP, GR designed the study, interpreted the data, and prepared the manuscript; SB was in charge of the Italian Mortality Data Base and participated to the record linkage phase of the study; SP, DS reviewed the death certificates and coded the causes of death; LDM, AZ, ADP carried out the record linkage procedure, data quality checks, and statistical analyses; LC, BS were in charge of the National AIDS Registry and participated to the record-linkage phase of the study. All authors contributed to a critical assessment of the manuscript, and they have read and approved the final version.

## References

[B1] CASCADE Collaboration Effective therapy has altered the spectrum of cause-specific mortality following HIV seroconversion AIDS 2000 20 741 749 10.1097/01.aids.0000216375.99560.a2 16514305

[B2] Palella FJ Baker RK Moorman AC Chmiel JS Wood KC Brooks JT Holmberg SD HIV Outpatient Study Investigators Mortality in the highly active antiretroviral therapy era. Changing causes of death and disease in the HIV outpatient study J Acquir Immune Defic Syndr 2006 43 27 34 10.1097/01.qai.0000233310.90484.16 16878047

[B3] Sackoff JE Hanna DB Pfeiffer MR Torian LV Causes of death among persons with AIDS in the era of highly active antiretroviral therapy: New York City Ann Intern Med 2006 145 397 406 16983127 10.7326/0003-4819-145-6-200609190-00003

[B4] Ledergerber B Furrer H Rickenbach M Lehmann R Elzi L Hirschel B Cavassini M Bernasconi E Schmid P Egger M Weber R Swiss HIV Cohort Study Factors associated with the incidence of type 2 diabetes mellitus in HIV-infected participants in the Swiss cohort study Clin Infect Dis 2007 45 111 119 10.1086/518619 17554711

[B5] D:A:D Study Group Use of nucleoside reverse transcriptase inhibitors and risk of myocardial infarction in HIV-infected patients enrolled in the D:A:D study: a multi-cohort collaboration Lancet 2008 371 1417 1426 10.1016/S0140-6736(08)60423-7 18387667 PMC2688660

[B6] Butt AA McGinnis K Rodriguez-Barradas MC Crystal S Simberkoff M Goetz Bidwell M Leaf D Justice C Veterans Aging Cohort Study HIV infection and the risk of diabetes mellitus AIDS 2009 23 1227 1234 10.1097/QAD.0b013e32832bd7af 19444074 PMC2752953

[B7] Pacheco AG Tuboi SH Faulhaber JC Harrison LH Schechter M Increase in non-AIDS related conditions as causes of death among HIV-infected individuals in the HAART era in Brazil PLoS ONE 2008 3 e1531 10.1371/journal.pone.0001531 18231611 PMC2211396

[B8] Crum NF Riffenburgh RH Wegner S Agan BK Tasker SA Spooner KM Armstrong AW Fraser S Wallace MR Triservice AIDS Clinical Consortium Comparisons of causes of death and mortality rates among HIV-infected persons: analysis of the pre-, early, and late HAART (highly active antiretroviral therapy) eras J Acquir Immune Defic Syndr 2006 41 194 200 10.1097/01.qai.0000179459.31562.16 16394852

[B9] Smit C Geskus R Walker S Sabin C Coutinho R Porter K Prins M CASCADE Collaboration Effective therapy has altered the spectrum of cause-specific mortality following HIV seroconversion AIDS 2006 20 741 749 10.1097/01.aids.0000216375.99560.a2 16514305

[B10] Selik RM Byers RH Jr Dworkin MS Trends in diseases reported on U.S. death certificates that mentioned HIV infection, 1987-1999 J Acquir Immune Defic Syndr 2002 29 378 387 11917243 10.1097/00126334-200204010-00009

[B11] Serraino D Zucchetto A Suligoi B Bruzzone S Camoni L Boros S De Paoli A Dal Maso L Franceschi S Rezza G Survival after AIDS in Italy, 1999-2006: a population-based study J Acquir Immune Defic Syndr 2009 52 99 105 10.1097/QAI.0b013e3181a4f663 19448558

[B12] Ancelle-Park R Expanded European AIDS case definition Lancet 1993 341 441 10.1016/0140-6736(93)93040-8 8094208

[B13] Conti S Masocco M Pezzotti P Toccaceli V Vichi M Boros S Urciuoli R Valdarchi C Rezza G Differential impact of combined antiretroviral therapy on the survival of Italian patients with specific AIDS-defining illnesses J Acquir Immune Defic Syndr 2000 25 451 458 10.1097/00042560-200012150-00011 11141245

[B14] Conti S Farchi G Galletti A Masocco M Napoli PA Pezzotti P Rezza G Toccaceli V Cariani G La notifica della mortalità per AIDS in Italia (1992): qualità della certificazione e sottonotifica Giornale italiano dell'AIDS 1997 8 12 16

[B15] Breslow NE Day NE Statistical methods in cancer research. Volume II: The design and analysis of cohort studies 1987 IARC Sci. Publ. N.82. Lyon: IARC 3329634

[B16] Serraino D Dal Maso L De Paoli A Zucchetto A Buzzone S Camoni L Suligoi B On changes in cancer mortality among HIV-infected patients. Is there an excess risk of death for pancreatic cancer? Clin Infect Dis 2009 49 481 482 10.1086/600823 19586399

[B17] Huxley R Ansary-Moghaddam A de Gonzalez Berrington A Barzi F Woodward M Type-II diabetes and pancreatic cancer: a meta-analysis of 36 studies Br J Cancer 2005 92 2076 2083 10.1038/sj.bjc.6602619 15886696 PMC2361795

[B18] Dal Maso L Polesel J Serraino D Lise M Piselli P Falcini F Russo A Intrieri T Vercelli M Zambon P Tagliabue G Zanetti R Federico M Limina RM Mangone L De Lisi V Stracci F Ferretti S Piffer S Budroni M Donato A Giacomin A Bellù F Fusco M Madeddu A Vitarelli S Tessandori R Tumino R Suligoi B Franceschi S Cancer and AIDS Registries Linkage (CARL) Study Pattern of cancer risk in persons with AIDS in Italy in the HAART era Br J Cancer 2009 100 840 847 10.1038/sj.bjc.6604923 19223894 PMC2653754

